# Correlation Between BMI and Severity of Acute Pancreatitis: A Retrospective Study

**DOI:** 10.7759/cureus.66917

**Published:** 2024-08-15

**Authors:** Neil Muscat, Firuza Soxibova, Naqqash Adnan, Ben Caruana Montaldo, Kholoud Abu Taha, Imran Alam, Oddai Alkhazaaleh

**Affiliations:** 1 General Surgery, Bolton NHS Foundation Trust, Bolton, GBR; 2 General Surgery, Royal Albert Edward Infirmary, Wrightington, Wigan and Leigh (WLL) NHS Foundation Trust, Wigan, GBR; 3 Internal Medicine, University of Malta, Msida, MLT; 4 General Surgery, Royal Bolton Hospital, Bolton, GBR; 5 Surgery, Wrightington, Wigan and Leigh (WLL) NHS Foundation Trust, Wigan, GBR

**Keywords:** systemic, local, complications, duration of hospital stay, obesity, severe, pancreatitis, acute, body mass index: bmi

## Abstract

Background

Pancreatitis, marked by sterile inflammation of the pancreas, can present as either acute or chronic. It involves the premature activation of proteolytic enzymes, leading to autodigestion, inflammation, and potential systemic effects. This study investigates the impact of obesity on the severity of acute pancreatitis, given its role in systemic inflammation and its association with severe morbidity and mortality.

Methods

A retrospective analysis was conducted on patients treated for acute pancreatitis over a five-month period at the Royal Albert Edward Infirmary, Wrightington, Wigan and Leigh (WLL) NHS Foundation Trust, Wigan, United Kingdom. Patients were evaluated using diagnostic criteria such as abdominal pain, elevated serum enzyme levels, and imaging results. The study explored correlations between BMI and pancreatitis severity, hospital length of stay, and complications, applying the Atlanta severity classification.

Results

The analysis revealed a weak, statistically insignificant correlation between BMI and the severity of acute pancreatitis, hospital stay length, and complications. This was consistent across various statistical methods, including Pearson correlation coefficients and multiple linear regression. These findings suggest that, while obesity may influence the inflammatory response in acute pancreatitis, it does not have a significant impact on clinical outcomes within this cohort.

Conclusions

The study highlights the complex role of obesity in exacerbating pancreatic inflammation but also emphasizes the need for larger, more definitive studies to explore this relationship further. It underscores the importance of early recognition and intervention in managing acute pancreatitis, regardless of BMI status.

## Introduction

Pancreatitis is a condition characterized by sterile inflammation of the pancreas, which can be subdivided into acute or chronic. This involves the premature activation of proteolytic enzymes produced by the pancreatic acini, which, in turn, causes autodigestion of the pancreas and an inflammatory response. The effects can be limited to the pancreas, but in some cases, they will spread to affect the rest of the body, causing systemic inflammation. Due to the risk of severe morbidity and mortality, there has been increased interest in research into the prognosticators of pancreatitis. Pancreatitis can be classified as either acute or chronic. Acute pancreatitis is a sudden onset of reversible inflammation, while chronic pancreatitis is a long-term, progressive disease that causes irreversible changes due to continual inflammation [1]. A diagnosis of acute pancreatitis can be made when two out of the following conditions are met: (1) abdominal pain in keeping with pancreatitis; (2) elevated serum amylase or lipase that is at least three times more than the upper limit; and (3) imaging showing pancreatic inflammation. The pain in pancreatitis is classically epigastric and radiates to the back, is sharp in nature, and is associated with nausea and vomiting. If the cause is biliary obstruction, then symptoms such as jaundice, dark urine, and steatorrhea may also be present.

According to the World Health Organization, obesity is a pathological state characterized by excessive accumulation of body fat and a BMI of ≥30 kg/m². Obesity is acknowledged as a chronic, low-grade inflammation that elevates the risk of numerous disorders, including cardiovascular and cerebrovascular diseases, type 2 diabetes, certain cancers, and acute pancreatitis [2]. Obese individuals’ fat tissue not only functions as a storage site for excess fat, but it also acts as an active endocrine organ that releases various pro-inflammatory signaling molecules, such as tumor necrosis factor alpha (TNF-α), IL-6, and CRP. These inflammatory cytokines contribute to the development of systemic inflammation, which can have a harmful impact on overall health [3]. Therefore, obesity plays a significant role in exacerbating the inflammatory cascade experienced during acute pancreatitis through a complex interplay of adipose tissue, cytokine release, and metabolic disturbances. The augmented severity of pancreatitis in obese individuals is attributed to heightened systemic inflammation, the localized impact of visceral fat, and metabolic dysregulation [3].

This study aims to investigate obesity’s role in increasing the severity of acute pancreatitis through this augmentation of the inflammatory cascade by statistically analyzing data obtained retrospectively. Additionally, the correlation between increasing obesity, measured as BMI, pancreatitis-related complications, and the length of hospital stay in the context of acute pancreatitis was also studied.

## Materials and methods

Between January 1, 2023 and May 31, 2023, 103 patients were treated for acute pancreatitis at the Royal Albert Edward Infirmary, Wrightington, Wigan and Leigh (WLL) NHS Foundation Trust, Wigan, United Kingdom. A total of 59 patients were included in this retrospective analysis, consisting of 22 females and 37 males. Comprehensive patient information was collected using a pre-made Excel document detailing age, sex, height and body weight, CT findings, ultrasound findings, magnetic resonance cholangiopancreatography findings, evidence of local or systemic complications, need for tertiary center transfer, etiology, mortality, and length of hospital stay. With this information, an Atlanta severity classification was calculated for each patient using the aforementioned criteria. BMI was calculated as weight in kilograms divided by height squared (kg/m²).

Inclusion and exclusion criteria

The study included patients ages 15-95 years who fulfilled the diagnostic criteria of acute pancreatitis, irrespective of causative etiology. Exclusion criteria encompassed chronic pancreatitis, pancreatic cancer, a history of pancreatic surgery, severe psychiatric disorders, and incomplete data.

Primary outcome

The primary outcome measure was a correlation between BMI and acute pancreatitis Atlanta severity.

Secondary outcomes

Secondary outcome measures included correlations between BMI, length of hospital stay, and pancreatitis-related local or systemic complications.

Statistical analysis

Statistical analysis was carried out utilizing the Pearson correlation coefficient to explore correlations between BMI and the severity of pancreatitis, length of hospital stay, and pancreatitis-related local or systemic complications. A p-value of <0.05 was considered significant. To account for differences in the number of males and females recruited, a two-tailed t-test (equal variances assumed, with normality analyzed using the Shapiro-Wilk test) was performed. Additionally, a multiple linear regression analysis was conducted separately to examine the influence of age, sex, and pancreatitis etiology on BMI.

## Results

**Figure 1:**
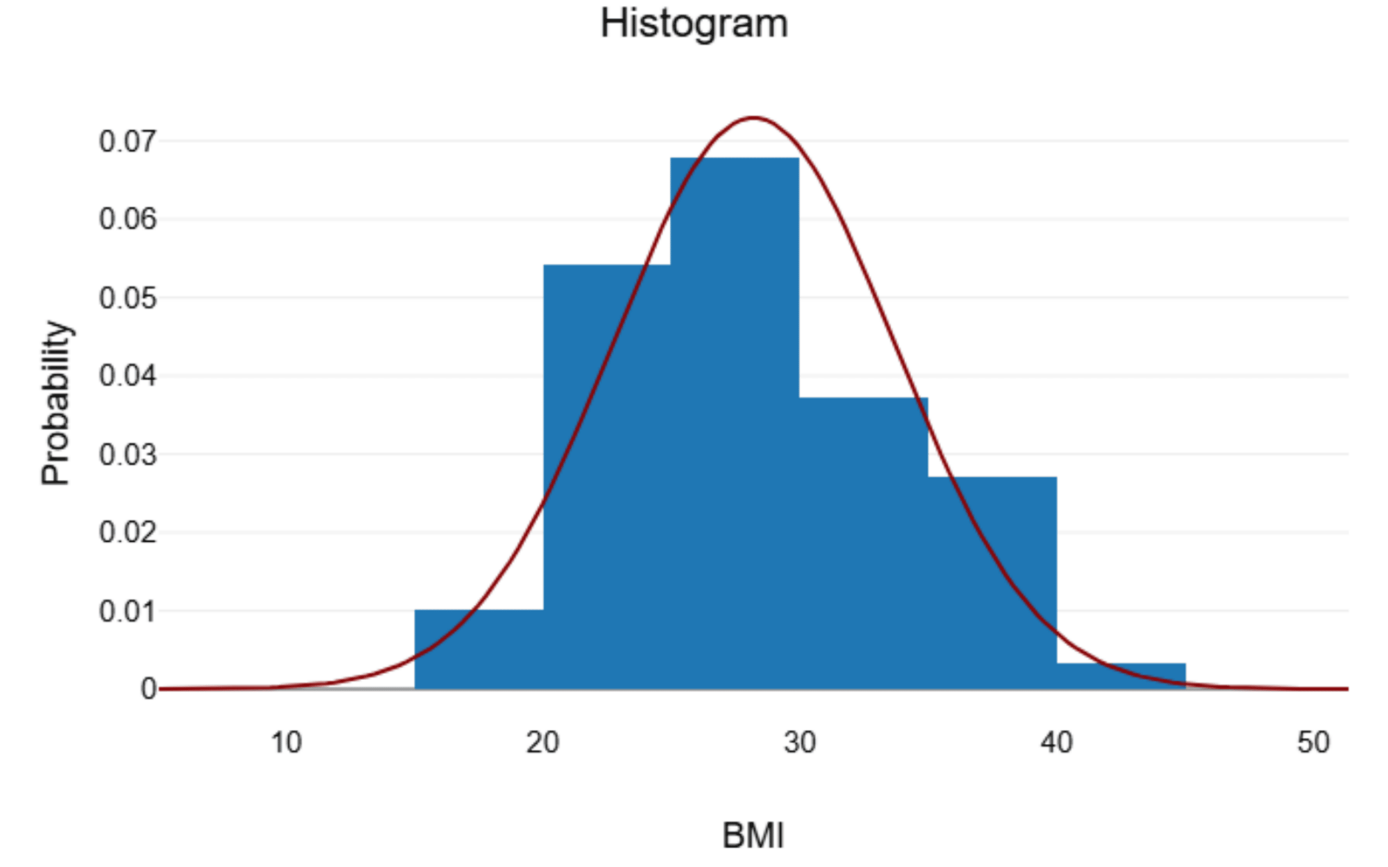
Histogram depicting the probability of various BMIs among participants

After applying the exclusion criteria, the study enrolled 59 patients, comprising 22 females and 37 males. The mean BMI of these participants was 28.2 (Figure 1).

The mean BMI of participants with mild pancreatitis, according to the Atlanta classification, was 28.45. Those with moderate-severity pancreatitis had a mean BMI of 26.15, while participants with severe pancreatitis had a mean BMI of 29.84 (Table 1).

**Table 1:** Frequency of pancreatitis severities according to the Atlanta classification, with corresponding BMI data for each group

Severity	Frequency	Mean BMI	Standard deviation	Minimum	Maximum
Mild	35 (59.4%)	28.45	5.35	17.9	41
Moderate	13 (22%)	26.15	6.59	15.1	39.4
Severe	11 (18.6%)	29.84	4.27	23.4	36.5

A statistical analysis using the Pearson correlation coefficient revealed an R-value of 0.0151 (p = 0.90) for the relationship between BMI and the severity of acute pancreatitis. Correlations between BMI and hospital stay, and BMI and complications, yielded R-values of 0.1632 (p = 0.22) and 0.1149 (p = 0.30), respectively.

A two-tailed t-test showed that the difference in BMI between male and female participants was not statistically significant, with t(57) = 1.31, p = 0.197, and a 95% CI of (-1.03, 4.88).

The regression model indicated that age, sex, and pancreatitis etiology explained 58.79% of the variance in BMI. ANOVA testing revealed this effect was not significantly different from zero, with F = 1.05, p = 0.426, and R² = 0.59 (Table 2).

**Table 2:** ANOVA for assessing the level of variability within the regression model

R	R^2^	Adjusted R^2^	Standard error of the estimate
0.77	0.59	0.03	5.66

## Discussion

The worldwide incidence of acute pancreatitis was 10-45 people per 100,000 inhabitants, while chronic pancreatitis affects 50-200 cases per 100,000 [4]. These figures vary based on geographical and societal factors. An increase of 3.07% per year of acute pancreatitis in the Western world over the previous few decades has also been noted [5], with mortality estimated to be as high as 20% in severe cases of the disease. Patients who suffer from chronic pancreatitis have a median survival of 15-20 years [6].

Pancreatitis has multiple risk factors, predominantly a biliary etiology, excessive alcohol consumption, and metabolic conditions like dyslipidemia and diabetes mellitus. Biliary lithiasis, forming stones that may obstruct the pancreaticobiliary junction or the ampulla of Vater, is a major cause, contributing to up to 40% of acute pancreatitis cases [7]. Alcohol, particularly in large quantities, can trigger inflammation through oxidative stress, affecting both acute and chronic forms of pancreatitis [8]. Less common causes include endoscopic retrograde cholangiopancreatography (ERCP), certain medications, and autoimmune conditions.

Complications from pancreatitis are substantial, impacting morbidity and mortality, and can be local or systemic. Local complications include pancreatic necrosis, which may require antibiotic treatment or surgery [9], and pancreatic pseudocysts, which may resolve spontaneously or cause further issues such as infections or obstructions. Systemic effects are driven by inflammatory mediators spreading through the body, potentially leading to systemic inflammatory response syndrome, organ failure [10], and severe outcomes like sepsis, which is a major cause of death in these patients [11].

Management of acute pancreatitis involves immediate fluid resuscitation per the NICE guidelines, intravenous fluids, and monitoring of vital parameters to ensure stabilization. Early enteral nutrition is recommended, unless contraindicated by symptoms like nausea or vomiting. Antibiotics are reserved for cases with confirmed infections, and ERCP is utilized to relieve biliary obstructions when identified [12]. Early and appropriate intervention is crucial to managing the complications associated with pancreatitis effectively.

Active treatment for acute pancreatitis is determined by the evolution of complications. In severe cases, supportive organ therapies such as hemofiltration for kidney failure and respiratory support for acute respiratory distress syndrome are essential. Significant hemorrhages may necessitate embolization or surgery. For infective pancreatic necrosis and sepsis, antibiotic and procedural interventions are required. Early surgical debridement of necrotic tissue typically worsens outcomes, leading to a preference for less invasive methods like percutaneous drainage as the primary treatment [13].

Acute pancreatitis is classified into mild, moderate, or severe categories according to the revised Atlanta classification. Mild cases are self-limiting, requiring only supportive care. Moderate cases may involve short-term organ failure or complications not leading to organ failure, whereas severe cases are marked by persistent organ failure lasting over 48 hours [14]. Severity is frequently assessed using predictive scores such as the Ranson criteria, Modified Glasgow Imrie score, SOFA, and Apache II, which consider factors like age, lab results including white blood cell count, lactate dehydrogenase, and levels of urea and glucose to evaluate the necessity of operative intervention and the likelihood of mortality [15].

The CT severity index is another tool used to grade pancreatitis severity on CT imaging. It includes the Balthazar score, which rates the extent of peripancreatic fluid collections and inflammation. This, combined with the percentage of necrotized pancreatic tissue, provides a composite severity score [16].

A number of studies have been conducted over several decades investigating the role of obesity in pancreatitis. A comparative study conducted in 1990 concluded that increasing weight simply increased the risk of early systemic complications but reported no direct positive correlation between obesity and the incidence of mortality or local complications [17]. The first meta-analysis investigating this correlation, published by Martínez et al. in 2004 [18], concluded that obesity significantly increases the risk of both local and systemic complications in acute pancreatitis. Following this, the same author also concluded that obesity increases mortality in the context of acute pancreatitis [19]. Further meta-analyses in recent years have specifically linked patients who are overweight (BMI >25) to an increased incidence of severe acute pancreatitis (SAP) and thus highlighted the importance of BMI as a prognostic indicator [20,21]. Of note, current literature suggests that waist circumference, peripancreatic visceral adipose tissue, and visceral fat-to-muscle ratio exhibit an even stronger correlation with SAP compared to simple BMI or body weight [22,23].

The primary outcome of this retrospective study was to investigate the relationship between increasing body weight and the severity of acute pancreatitis. The data suggests a statistically insignificant positive correlation between these two variables. The data pertaining to the secondary outcomes investigating the relationship between increasing body weight, hospital stay, and local or systemic complications in the context of acute pancreatitis also suggested a statistically insignificant positive correlation between the variables. These conclusions were further analyzed utilizing a regression model to account for several other variables, including age, sex, and the etiology of individual acute pancreatitis etiology. An ANOVA concluded that the effect of these variables on the primary and secondary outcomes was not statistically significant.

In the context of acute pancreatitis, the secretion of the aforementioned pro-inflammatory cytokines through the endocrine function of adipose tissue worsens both local and systemic inflammatory reactions. Obesity is associated with a heightened inflammatory state, contributing to prolonged and more severe episodes of acute pancreatitis through orchestrating a shift from an anti-inflammatory M2 macrophage phenotype to a pro-inflammatory M1 phenotype [24]. Through crucial encouragement of the activation and infiltration of inflammatory cells like macrophages and neutrophils into the pancreatic tissue, cytokines such as TNF-α and IL-6 play a pivotal role in the development and maintenance of the inflammatory response typically experienced during acute pancreatitis. The recruitment, activation, and influx of inflammatory cells result in an increased generation of reactive oxygen species, which, in turn, induces additional cytokine release, establishing a vicious cycle of inflammation and tissue injury. In obese patients, a greater amount of free fatty acids (FFAs) is available to be broken down from adipose tissue, which further increases the severity of pancreatic inflammation owing to their cytotoxic properties. FFAs can also independently trigger the production of fatty acid ethyl esters, which are harmful compounds that directly damage pancreatic acinar cells, resulting in cell death and further inflammation. Visceral fat, which envelops internal organs such as the pancreas, significantly contributes to this inflammatory cascade. In acute pancreatitis, visceral fat adjacent to the pancreas secretes inflammatory mediators directly adjacent to pancreatic tissue, contributing to the augmentation of severe local inflammation and tissue damage. Moreover, the extensive infiltration of immune cells into this visceral fat causes complications such as infection and necrosis of adipose tissue. Apart from accentuating the inflammatory cascade, obesity also prevents its resolution through metabolic dysregulation such as insulin resistance and hyperglycemia. These disturbances negatively impact the immune system’s ability to heal and recover from such insults [25-28].

Further extensive research is required to address the limitations related to the sample size and the variability of clinical presentations, including patient co-morbidities and etiology, in order to elucidate the full extent of the impact of obesity on acute pancreatitis. These studies should focus on enhancing predictive models and refining management strategies to improve clinical outcomes for this potentially severe and life-threatening condition. By gaining a deeper understanding of the complex relationship between obesity and pancreatitis, healthcare providers can develop more effective interventions and potentially reduce the adverse outcomes associated with this intricate interaction.

## Conclusions

This comprehensive examination and retrospective analysis underscore the complex relationship between obesity and acute pancreatitis, highlighting how obesity exacerbates the severity of this inflammatory condition. Although the study found a weak, statistically insignificant correlation between BMI and the severity of acute pancreatitis, it is clear that obesity influences the inflammatory processes in the pancreas. Adipose tissue releases pro-inflammatory cytokines, contributing to a systemic inflammatory state that can worsen pancreatitis and complicate clinical outcomes.

The findings emphasize the need to view obesity not just as a risk factor but as a significant contributor to the pathophysiology of acute pancreatitis. Obese patients are more likely to experience severe complications and prolonged inflammatory responses, driven by complex metabolic and immune mechanisms. The study also highlights the importance of developing targeted therapeutic strategies to address the specific challenges obesity presents in managing pancreatitis.
